# Autonomous Mutual Authentication Protocol in the Edge Networks

**DOI:** 10.3390/s22197632

**Published:** 2022-10-08

**Authors:** Ruey-Kai Sheu, Mayuresh Sunil Pardeshi, Lun-Chi Chen

**Affiliations:** 1 Department of Computer Science, Tunghai University, Taichung 407224, Taiwan; 2 AI Center, Tunghai University, Taichung 407224, Taiwan

**Keywords:** authentication protocol, autonomous systems, security, secure edge networks, resource-constrained devices

## Abstract

A distinct security protocol is necessary for the exponential growth in intelligent edge devices. In particular, the autonomous devices need to address significant security concern to function smoothly in the high market demand. Nevertheless, exponential increase in the connected devices has made cloud networks more complex and suffer from information processing delay. Therefore, the goal of this work is to design a novel server-less mutual authentication protocol for the edge networks. The aim is to demonstrate an autonomous mutual authentication amongst the connected smart devices within the edge networks. The solution addresses applications of autonomous cars, smart things, and Internet of Things (IoT) devices in the edge or wireless sensor networks (WSN), etc. In this paper, the design proposes use of a public-key system, octet-based balanced-tree transitions, challenge–response mechanism, device unique ID (UID), pseudo-random number generator (PRNG), time-stamps, and event specific session keys. Ultimately, server-less design requires less infrastructure and avoids several types of network-based communication attacks, e.g., impersonating, Man in the middle (MITM), IoT-DDOS, etc. Additionally, the system overhead is eliminated by no secret key requirements. The results provide sufficient evidence about the protocol market competitiveness and demonstrate better benchmark comparison results.

## 1. Introduction

In edge networks, local data processing and storage helps to make it independent of complex network infrastructure [1,2]. Therefore, edge network devices should be protected to avoid major attacks [3,4] such as DDOS, ransomware, man in the middle (MITM) attack, etc. As the paradigm of distributed computing, edge network devices centralize data centers and acts as smart things to overcome cloud computing limitations. High-speed data networks such as 5G wireless communication have boosted the application of edge devices and increased the vulnerability at the same time. Hence, this work uses authentication protocol to secure the multiple edge device interconnectivity for the Internet of Things (IoT) communication.

Evolution of edge computing marks a significant change by overcoming the client–server or network architecture limitations. As the edge devices can function independently, a network communication delay and shortest path planning is eliminated. Therefore, for the secure communication of the edge devices we have designed an autonomous mutual authentication protocol. Security is an essential factor for protecting the data confidentiality and integrity. To ensure secure communication, this work provides authentication within the independent edge devices. The scope for this work is providing a security solution for the protection of IoT devices against malicious contents to function smoothly and efficiently. The data communication consists of a unique session key generation and exchange within edge devices to achieve mutual authentication by the protocol. The background knowledge for this work contains authorization, public-key cryptography, challenge–response mechanism, session keys, PRNG, and mutual authentication. Public-key cryptography is also popularly known as asymmetric cryptography, which uses unique pairs of different keys for encryption and decryption as public key and private key, respectively. These keys are generated as a part of cryptographic algorithms by a one-way function in which the public key is disclosed to everyone for communication. The authorization of the official registered user is said to be granted or approved after the genuine user permission is assigned, e.g., Diffie–Hellman key exchange, elliptic-curve cryptography, Rivest–Shamir–Adleman (RSA) algorithm, etc. In the challenge–response mechanism, the party wishing to communicate first needs to provide a valid answer to the question as set by the other party for authentication. The session keys are part of communication protocol, which are shared for the validity confirmation between multiple parties as a part of the agreement. The PRNGs are derived from linear congruential generator algorithms, which function as a deterministic random bit generator having properties similar to the approximation of a random number. Mutual authentication can be defined as an authentication protocol process performed between the two parties at the same time.

The motivation for this work is given as “How to design a mutual authentication protocol for the autonomous devices in the edge network?” [5,6,7,8]. Considering the independence feature of the autonomous devices for mutual authentication, they must not be restricted to a particular area network after the registration phase [9,10]. Therefore, referring to the autonomous vehicular protocol design community, many researchers have started improving the security authentication and confidentiality communication, following Vamsi Paruchuri, Arjan Durresi, Rajgopal Kannan, and S. Sitharama Iyengra in 2004, who applied it in autonomous system traceback for authentication [11]. The inclusion of third-party infrastructure in the authentication of the autonomous vehicle process has been a challenge that acts as an overhead on the resource-constrained IoT devices, which needs to be resolved.

Henceforth, in this work, a novel challenge–response model using pseudo-random-number-based octet’s transitions is proposed. Finally, the results have demonstrated to be an efficient protocol in the edge network and its respective performance details.

### 1.1. Objectives

Authentication is considered to be one of the key components for performing secure communication within distributed systems. Therefore, it must be included within the recent autonomous edge networks for better security. There are multiple authentication protocols in the distributed system, but the process is incomplete without servers. Therefore, this work proposes a novel protocol that can authenticate multiple devices by mutual authentication without the need of any server for initiation, authentication, and management.

- A novel protocol design for the mutual authentication in the edge networks: This work demonstrates an idea for the challenge–response model by establishing an authentication process within the autonomous devices performing the session key exchange as the part of valid response by the requesting party. Therefore, mutual authentication is achieved after solving challenges, which are provided by both the parties to each other. The protocol design has included use of a unique session key, different time-quarter-based PRNG random values, time-stamps, and the transitions within the group of octet’s position.
- Server-less mutual authentication within the independent devices: Authorization is selected as the basic requirement for this work. Thus, authorized devices can only initiate and implement the authentication protocol. Autonomous operations make the multiparty authentication independent of the external server. Thus, the traditional approach of using server or third-party systems within the mutual authentication protocol limitations is resolved by this work. Therefore, complete autonomy on the operations is achieved.
- Multiple IoT device authentications within the network: Multiple IoT devices exist within a network with the mobile workstation as a portable device. This work presents the communication within the autonomous edge devices that can iterate, process, and authenticate each other without any fixed system/server for achieving complete autonomous status. Successively, autonomous devices with public keys can mutually authenticate each other and can form a trusted network. The IoT devices can connect to multiple devices within the wireless local network for a single communication session.
- No additional support of infrastructure and IoT resource-constrained device utilization: Architecture of several authentication protocols consists of a service server for key distribution, ticket-granting server (TGT), for process validation and authentication server for confirming the process and declaring successful authentication. Inclusion of many servers creates a bottleneck in the system. Additionally, several calculations with different servers are not suitable for IoT resource-constrained devices. In this work, there is no need for a ticket-granting (TGT), service server and authentication server. Whereas, the need for registration server after registration phase is completely eliminated. Minimal calculations and no secret key leads to reduced overhead on resource-constrained devices.

### 1.2. Applications

- Secure Autonomous Cars: Recently, many car manufacturing companies are competing in the market to provide self-driving/autonomous cars and vehicle platooning. The connectivity within cars for exchanging information is authenticated for secure communication.
- Secure Drones: A drone network is usually required for smart farming; package delivery for food, products, medical vaccines at remote or higher altitude places; tracking lost people on mountains; synched drones for entertainment; etc.
- Secure Satellite Communication: The satellite network for providing region/countrywide internet access needs to be synchronized and interconnected. New satellites can join and later can reconnect using an autonomous mutual authentication protocol. Thus, dependency on third parties is reduced.
- Secure IoT and Device Communication: All the end devices within a network can connect to each other securely by authentication, i.e., IoT devices, laptop, computer, tablet, etc. The communication between these authenticated devices is secured by cryptography and has a dynamic session key instead of using a master secret key.

The description of this work is organized herein as follows: presentation of literature survey in Section 2; protocol phases as methodology in Section 3; and theorems with proofs in Section 4. Afterward, the protocol verification logic is given in Section 5 and protocol defense in Section 6. Lastly, the experiments are given in Section 7 followed by the conclusions.

## 2. Literature Survey

The privacy preservation in the autonomous transportation system is presented by Sucasas V. et al. [2], and similarly for Huang et al. [12]. This work focuses on eliminating the pseudonym-based congestion and trusted authority dependency. Thus, the autonomous protocol to reduce dependency on trusted authority is implemented using bilinear maps, elliptic curve discrete logarithm problem (ECDLP), collusion attack algorithm (CAA), fiat-shamir heuristics, and hash chains. The autonomous connectivity between multirobot systems is demonstrated by Wei Liang et al. [13]. This scheme uses trusted identity authentication and the hash-pool-based consensus algorithm. The operations include permuting hash functions in hash pool, random number generator, device IDs, public keys, private keys, and multiple signatures. The JTAG authentication using an autonomous algorithm is presented by Lapeyre S. et al. [14]. This work has a lightweight plug-and-play solution for automated test equipment with two cryptographic hashes algorithms and claims to be better than the SHA3 algorithm. An autonomous protocol for distributed IoT security by smart contract is demonstrated by Wickström J. et al. [15]. This work design consists of an ethereum smart-contract-based security model while keeping it independent of network connections and acts as a generic task creator with reporting. The peer-to-peer (P2P) autonomous authentication scheme is presented by Alkhalaf S. [16]. The anonymous access problem is resolved by using group identification and support vector machine-based classification. In a decentralized autonomous network, a blockchain-based authentication is demonstrated by Wang M. et al. [17]. This work overcomes the complexity of cross-domain authentication by using multicertification authority (CA) and a distributed blockchain base for gaining trust. The artificial neural group key synchronization-based security within autonomous vehicles is presented by Khan M.Z. et al. [18]. The architecture consists of a vehicle-to-everything (V2X) heterogeneous network for information fusion, synchronization using ring framework, B-tree, and triple layer tree parity machines for key exchange processes. The device-to-device (D2D) security by multichannel authentication is demonstrated by Li T. et al. [19]. The Diffie–Hellman key exchange is used within optical-link communication with an LED light and camera in a D2D multichannel authentication having full and half duplex modes. The autonomous robot communication security within the shipping network is presented by Yang J. et al. [20]. The robots are used for parcel delivery using QR codes, hash functions, and asymmetric encryption, whereas, the Siamese network performs noncooperative user identification and re-identification. The autonomous vehicle applications in smart farming are presented by Bilbao-Arechabala S. et al. [21].

The complete automation of smart farming is achieved by ISO 7798-2 security specifications—heterogeneous swarms are used in cloud middleware to operate drones, autonomous vehicles, etc. Autonomous-vehicle secure connectivity using IoT devices and key management framework is demonstrated by Jha S. et al. [22]. The vehicular network authentication is performed by blockchain based on hash graphs that can perform thousands of transactions per second and a framework designed using batch rekeying and logical key hierarchy (LKH). The 5G cooperative autonomous connectedness and driving is presented by Bagheri H. et al. [23]. This system uses 5G-based extensive authentication protocol (EAP) supporting 3GPP and non-3GPP communication networks, independent access, and mobility management function with session management function. The autonomous communication within the P2P network is demonstrated by Rahmani L. et al. [24]. A distributed hash table for agent lookup is shared by all the communicating agents and uses public-key cryptography for secure P2P communication with end-to-end encryption. An IoT mutual authentication protocol for Things-To-Things (T2T) is presented by Lounis K. et al. [25]. The T2T protocol uses physical unclonable functions (PUFs) with dual-level-challenge response pairs for the IoT authentication. V2X communication-based efficient authentication for protection against DDOS is demonstrated by Ko T. et al. [26]. The V2X system uses a security credential management system (SCMS), which classifies multiple similar messages in different categories for authentication and uses advanced verify-on-demand (AVoD) for signature verification with threat analysis. An improved isolation forest method for autonomous-vehicles-attack detection is presented by X. Duan et al. [27]. The detection of data-tampering attack is performed here using data mass and scoring for anomaly detection as a part of intrusion detection. An autonomous vehicle smart-parking system with the fog–blockchain architecture is presented by Shahzad A. et al. [28]. Smart parking helps to recognize the parking location with the help of fog nodes to IoT, the proof-of-concept by lightweight blockchain and a cryptographic module is utilized. Blockchain-based autonomous vehicle platoon management in 5G is demonstrated by Wu B. et al. [29]. This real-time system improves traffic management with public-key cryptography and 5G-enabled revocable attribute-based encryption (RABE) with key distribution and revocation. P2P drone communication using blockchain is presented by Kumar M.S. et al. [30]. The drone base communication uses blockchain with GPS coordinates to avoid spoofing attacks and keeps the blacklisted database.

### Analysis of the Survey Limitations

- The need for additional infrastructure for the authentication protocol: Several recent works that are developing authentication protocols include blockchain-based operations by having dependency on the service server, ticket-granting server (TGT), and authentication server. Therefore, these authentication protocols are not suitable for the autonomous devices as they require higher dependency on the multiple systems for the purpose of authentication.
- High-calculation requirements for the IoT resource-constrained devices: The traditional cryptographic algorithms and protocols are not suitable for the autonomous devices as most of the IoT resource-constrained devices possess limited memory and processing power. Therefore, design of a new authentication process is required to avoid high calculations on the IoT devices and to perform efficiently for multiple authentications.
- Design issues limiting the protocol performance: The inclusion of popular technologies and references, i.e., blockchain, Kerberos, elliptical curve cryptography (ECC), in the protocol design without a specific objective is one of the large mistakes in many works. Such design issues lead to low performance and bottlenecks within the system, which are not suitable for the autonomous devices.

## 3. Methodology

**Figure 1:**
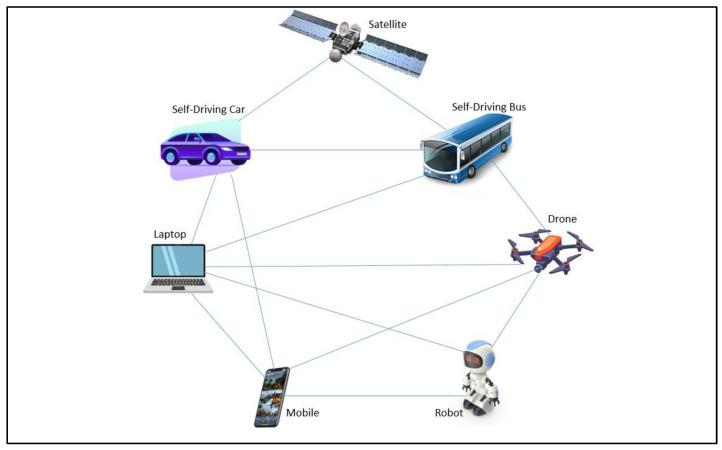
Autonomous device connectivity.

The architecture for autonomous-device connectivity in general is shown in Figure 1. The autonomous devices can connect 1:1, 1:M (many), and M:M device connections, whereas autonomous devices can operate independently or collectively. The purpose of connectivity is to receive status, position, information exchange, and control remote devices. The applications are given as drone-based delivery, robotic fire extinguishers, drone base smart farming, self-driving car/bus, car platooning, smart surveillance, etc.

### 3.1. Initialization Phase

The purpose of the initialization phase is to define the structural setup required for protocol functioning. All the citizens interested in securing their personal devices can download the autonomous protocol setup by registering on the government’s national website to utilize this service. The distributed network connecting every state/region’s government server will also keep the record logs for the public keys with active and migrated registered devices. Therefore, the registered user’s device information will be kept on both national and regional servers to allow for ease of interoperability.

### 3.2. Registration Phase

The users follow the instruction for the edge device registration on the national portal *RS*. The user’s personal unique ID (UID) and device ID are required to complete the registration process on the national website. Each device is assigned a unique public key that can be an automobile car, a computer/laptop, autonomous drones, ships, robots, etc. The user will also receive the public keys of all the registered devices within that state/region. Henceforth, the regional-level portal server will possess a list of local registered edge devices, which will be accessible while traveling to other regions and can access new public keys without the need to re-register his/her devices. The national central server will also possess the state wise edge devices public key’s, which are accessible to the trusted authorities. A distributed database stores the UID linked device details in either cloud or blockchain based server because of immutability and distributed ledger feature.

### 3.3. Authentication Phase

**Figure 2:**
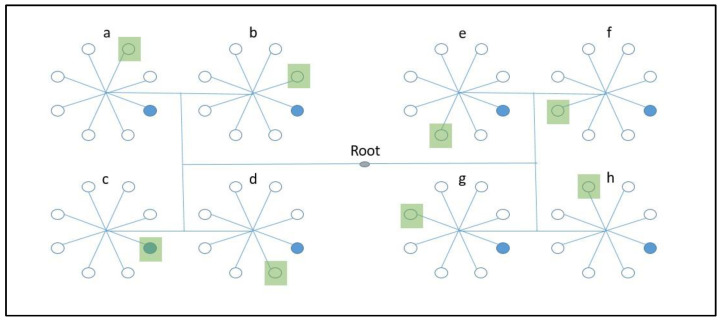
Octet-based balanced tree.

The public key and protocol interaction format received by user C makes him or her eligible for the authentication process. Figure 2 presents the balanced tree containing 8 octets. The PRNG is applied here in the four time-quarters based on each six-hour slot. For every time-quarter, the PRNG parameters are changed and the pseudo-random numbers generated are distributed serially in the 8 octets. According to Figure 2, the sender initiates the protocol by sending the time-stamp as a challenge in the message, where the time-stamp’s last value or a random number is taken as $node_{value}$. The number obtained can be seen in the figure; blue highlights the position in all adjacent octets, as given in Equation (1).

(1) $$node_{value}=\overset{7}{\underset{k=0}{\sum }}(node_{value}+8)$$

Therefore, the first challenge set by the sender is completed after the response is combined with the selected pseudo-random values by XOR, and responds to it in the second message. In the second challenge, which is set by the responder, the XOR of the consecutive octets is taken at a particular position by incrementing itself every time in Equation (2), as shown by green in Figure 2.

(2) $$node_{value}=\overset{7}{\underset{k=0}{\sum }}(node_{value}+9)$$

**Figure 3:**
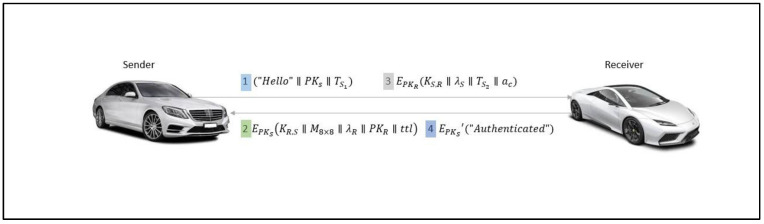
System model.

Figure 3 presents the mutual authentication protocol process within the autonomous sender and receiver with notations from Table 1. 

The detailed stepwise process for autonomous mutual authentication follows.

(1) The sender *S* is required to initiate the authentication process by providing the “Hello” message with his public-key $PK_{s}$ and the current time-stamp $T_{S_{1}}$ in seconds. The $PK_{s}$ provided by the sender is actually a part of challenge 1 sent for the octet node to be selected.
  $$S\to R:\left(“Hello”∥PK_{s}∥T_{S_{1}}\right)$$
(2) The receiver R first checks for the validity of the sender’s public key $PK_{S}$ and takes the last value of the time-stamp $T_{S_{1}}$ sent by the sender S with modulus 8. The receiver then sets the PRNG parameters based on the time-quarter and generates the pseudo-random numbers for 8 octets. Therefore, the value obtained from the time-stamp with modulus 8 of the sender is taken to select the first octet’s value and the same value from consecutive octets. Successively, the combination by average from Equation (1) obtained from random numbers is a solution to the first challenge and is returned to the sender for confirmation as transition $\lambda_{R}$. The second message is formed by the octate group as $M_{8\times 8}$ multidimensional matrix, $\lambda_{R}$ transition value, $PK_{R}\;$ as receiver’s public key, ttl as time-to-live for this message’s validity, and $K_{S,R}=H\left(\lambda_{R}⊕\;T_{S_{1}}\right)\;$ as the session key, which is encrypted by the public key of sender $E_{PK_{S}}$.
  $$R\;\to S:\;E_{PK_{S}}\left(K_{R,S}∥M_{8\times 8}∥\;\lambda_{R}∥PK_{R}∥ttl\right)$$
(3) The sender, after receiving the response, decrypts it by his private key $D_{SK_{S}}$. Optionally, the sender then checks the transition value $\lambda_{R}$, and calculates it from the consecutive octates. Thus, the hash calculation of the session key $K_{R,S}$ achieves the challenge 1 confirmation. Challenge 2 is initiated by the next value by the previous challenge in the first octet, and then collects the value incremented every time in the consecutive octets, as shown by green in Figure 2. The transition value obtained by XOR is $\lambda_{S}$. Successively, the session key $K_{S,R}=\left(\lambda_{S}⊕\;T_{S_{2}}\right)$ with the current time-stamp is calculated and sent to the receiver as the challenge 2 with device address. It is encrypted by the public key of receiver $PK_{R}$. This message has to be responded to before the time-to-live ttl given by the receiver.
  $$S\to R:\;E_{PK_{R}}\left(K_{S,R}∥\;\lambda_{S}∥T_{S_{2}}∥a_{c}\right)$$
(4) The receiver decrypts the received encrypted message by his private key $D_{SK_{R}}$ and obtains the challenge 2 response by the sender. The decrypted message is confirmed to be correct by calculating the transition value $\lambda_{S}$ and by the hash value of the session key $K_{S,R}$. The device address $a_{c}$ received is kept for the purpose of device identification. The final session key is $E_{PK_{S}}{}^{\prime }=H\left(K_{R,S}⊕K_{S,R}\right)$. This session-key-combining process is already known by the sender; when he or she decrypts the final message with a symmetric key to know about authentication from the receiver, then the mutual authentication is succeeded.
  $$R\;\to S:\;E_{PK_{S}}^{\prime }\left(“Authenticated”\right)$$

### 3.4. Communication Phase

The autonomous devices attempt to connect to the local devices dynamically with the purpose of instructing or information sharing. Subsequently, in the edge network, the devices are not mandatorily required to possess an internet connection or otherwise connect by Bluetooth and can request to connect by authentication with a condition of maximum two challenge–response trials. The autonomous authentication process is initiated only if the public key of the sender and receiver are present within the public-key database. Therefore, absence of such a public key indicates updating the public-key database of the communicating parties and later confirms their legitimacy.

### 3.5. Revocation Phase

Once the multiple autonomous devices authenticate each other and, if due to location/communication lag, the mutual session key is terminated. In the case of re-initiating the authentication, the autonomous protocol should be succeeded in the next two attempts; otherwise it will be blocked for one day. Every device maintains its own list of blocked devices which is cleared every day. The purpose of the key revocation phase is to keep log records for security audit at a regular interval.

## 4. Analysis of Hardness of Autonomous Protocol

**Theorem** **1.** *If the multiple communicating parties as sender* S *and receiver* R *can successfully complete the authentication process, in such cases the validating receiver* R *always accept the sender* S *as valid.*

**Proof** **of** **Theorem.** According to the autonomous protocol process with reference to Section 3.3 Authentication Phase. If the sender S and receiver R are equipped with the authentication process with the secret of octet-group balanced tree, key generation, cryptographic algorithm, time-quarters, challenge–response within the multiple parties, then the challenge is initiated by the receiver R as

$$R\;\to S:\;E_{PK_{S}}\left(K_{R,S}∥M_{8\times 8}∥\;\lambda_{R}∥PK_{R}∥ttl\right)$$

Therefore, $S\to R:\;E_{PK_{R}}\left(K_{S,R}∥\;\lambda_{S}∥T_{S_{2}}∥a_{c}\right)$ is confirmed to be valid when the receiver combines the temporal session key to be the final session key as $R\;\to S:\;E_{PK_{S}}{}^{\prime }\left(“Authenticated”\right)$. Ultimately, the successful completion confirms the authentication. □

**Theorem** **2.** *Considering the constructed possible solutions for the two challenges of octet-group balanced-tree transitions by the malicious user* ${\hat{M}}_{u}$ *, while assuming he is highly capable as receiver R. When* ${\hat{M}}_{u}$ *impersonated R and initiates the autonomous protocol process to approach S that he is real R, in such case the probability of* ${\hat{M}}_{u}$ *success is quite high.*

**Proof** **of** **Theorem.** The search for a solution is intractable by the zero-knowledge proofs (ZKP) process for the transitions in octet-group balanced tree. Therefore, the possible solutions to this problem are equivalent to computing matrix multiplications by the number theory concepts. For challenge 1, ${\hat{M}}_{u}$ choses a subtree to perform possible transitions during the attack. In every case of the challenge event, it is worth noting that the PRNG generates different parameters based on pseudo-random numbers, which are combined as ⊕ with a time-stamp to generate session keys for all the autonomous edge devices. Thus, ${\hat{M}}_{u}$ required for challenge 1 is to provide accurate tree nodes and its respective transition combination as the solution.

$$R\;\to S:\;E_{PK_{S}}\left(K_{R,S}∥M_{8\times 8}∥\;\lambda_{R}∥PK_{R}∥ttl\right)$$

For challenge 2, ${\hat{M}}_{u}$ needs to achieve the octet-based transition related to time-stamp-based parameters. Multiple octet values are stored as a transition, which is combined as ⊕ with the time-stamp as the final shared session key required to be calculated within a time limit.

$$S\to R:\;E_{PK_{R}}\left(K_{S,R}∥\;\lambda_{S}∥T_{S_{2}}∥a_{c}\right)$$

□

**Theorem** **3.** *Autonomous system authentication is a ZKP protocol*.

**Proof** **of** **Theorem.** In the autonomous protocol process, the temporal session keys $K_{R,S}$ and $K_{S,R}$ exchange are encrypted by the public keys of both the parties that are part of the challenge–response scheme. Consecutively, the use of pseudo-random numbers, transitions values, octet-based balanced tree, and time-stamp combination makes the protocol process very hard to analyze and construct an accurate solution. Ultimately, it can be noted that ${\hat{M}}_{u}$ is unable to devise a time-quarter-based solution and guess about any possible solution either for the challenge or ZKP. Henceforth, a strong claim for autonomous system authentication in the edge network is a ZKP protocol. The autonomous authentication achieves the ZKP process between multiple devices and can defend ${\hat{M}}_{u}$ ’s impersonation attack. □

## 5. Protocol Verification Logic

### 5.1. Message Exchange

The process below signifies the message exchange between the sender and receiver:

$$A\to B:\;K_{a},T_{1}\;B\to A:\;{\left\{K_{b,a},M_{8\times 8},\lambda_{b},K_{b},T_{s}\right\}}_{K_{a}}\;A\to B:\;{\left\{K_{a,b},\lambda_{a},T_{2},P_{a}\right\}}_{K_{b}}\;B\to A:\;{\left\{\right\}}_{K_{ab}^{\prime }}$$

This process notations can be elaborated as $K_{a,b}$ and $K_{b,a}$ as session keys for the protocol process, and $K_{ab}^{\prime }$ as an event session key with limited validity used before and after the protocol authentication with cryptography. During the protocol process initiation, the public key and time-stamps of sender A are used. Later, receiver B starts challenge 1 with public keys as temporal session keys $K_{ab}^{\prime }$ in the successive steps. The challenges solved by both the communicating parties and approved response results in the last step of protocol success as authentication.

### 5.2. Idealized Protocol

The construction of the idealized protocol is given below:

$$A\to B:\;\overset{K_{a}}{\to }A,T_{1}\;B\to A:\;{\left\{A\overset{K_{b,a}}{↔}B,M_{8\times 8},\lambda_{b},\overset{K_{b}}{\to }B,T_{2}\right\}}_{K_{a}}\;A\to B:\;{\left\{A\overset{K_{a,b}}{↔}B,\lambda_{a},T_{3},P_{a}\right\}}_{K_{b}}.\;\;B\to A:\;{\left\{\right\}}_{K_{ab}^{\prime }}.\;$$

The idealized protocol emphasizes the entities and the use of public keys amongst them for sharing with cryptographic operations. This protocol format is quite similar to the message exchange. Messages 1 and 2 contain the exchange of public keys. The cryptographic operations by public keys are performed in messages 2 and 3. The temporal-shared session key valid for an event is shared for confirmation in message 4 as $K_{ab}^{\prime }$.

### 5.3. Protocol Analyzed

The construction of the analyzed protocol according to the formal logic is given as follows:

(3) $$A\;believes\overset{K_{a}}{\to }A,\;B\;believes\;\overset{K_{b}}{\to }B\;A\;believes\;\left(S\;controls\;\overset{K}{\to }B\right)\;B\;believes\;\left(S\;controls\;\overset{K}{\to }A\right)\;A\;believes\;fresh\;\left(K_{a,b}\right),\;B\;believes\;fresh\left(K_{b,a}\right)\;A\;believes\;\left(A\overset{K_{a,b}}{↔}B\right)\;B\;believes\;\left(A\overset{K_{b,a}}{↔}\;B\right)\;B\;believes\;fresh\;\left(K_{ab}^{\prime }\right)$$

The protocol-analyzed formal logic given above presents the ownership of public keys by the users. Successively, the registration server is responsible for creating and assigning the public keys to users A and B, which are believed by both users to be correct. Server S is capable of generating genuine public keys and distributing it to both parties. Therefore, both the parties believe that fresh temporal session keys are exchanged amongst them for every new authentication process, and the final session key received by B, described in Section 3.3, authentication phase step 4, confirms the successful completion of the authentication process.

### 5.4. Final Beliefs

The conclusion of the final analysis is given by the final beliefs, as follows.

$$A\;believes\overset{K_{b}}{\to }B\;B\;believes\;\overset{K_{a}}{\to }A\;A\;believes\;A\overset{K_{ab}^{\prime }}{↔}B,\;B\;believes\;A\overset{K_{ab}^{\prime }}{↔}B\;A\;believes\;B\;believes\;A\overset{K_{ab}^{\prime }}{↔}\;B\;B\;believes\;A\;believes\;A\overset{K_{ab}^{\prime }}{↔}\;B$$

In this part of belief, the entities believe that the other entities are aware of their public keys. Ultimately, the final session key is known to both entities. Therefore, all the entities believe that the temporal session keys exchanged previously amongst them are trustable, which completes the mutual authentication process successfully.

## 6. Autonomous Protocol Defense for Attacks

- Impersonation: In case of autonomous authentication, a malicious user ${\hat{M}}_{u}$ needs to validate himself or herself to the server or third-party system by spoofing his identity. Therefore, ${\hat{M}}_{u}$ can directly take the identity of some valid user and can try to validate $S\;\to R:\left(“Hello”∥PK_{s}∥T_{S_{1}}\right)$. in the first message. If the identity belongs to an interstate user and he or she is not updated in the current state/region’s user list, then he or she will be rejected. Nevertheless, bypassing the identity validation, ${\hat{M}}_{u}$ will not be helpful to possess the authentication protocol steps to move further.
- Wormhole: ${\hat{M}}_{u}$’s presence can cause it to reroute the packets from different systems. In such a case, the sender can bypass step 1 but will fall short of the time to live (ttl) in protocol step 2. In step 2, the challenge 1 with $R\;\to S:\;E_{PK_{S}}\left(K_{R,S}∥M_{8\times 8}∥\lambda_{R}∥PK_{R}∥ttl\right)$ is initiated, which provides the required group of parameters in the encrypted message. Even though ${\hat{M}}_{u}$ can receive the message, unaware of the encryption and ttl, he or she will be rejected and blocked after two unsuccessful attempts.
- Sinkhole: In case of selective modification performed by ${\hat{M}}_{u}$, the autonomous protocol will be discontinued due to the hash-function calculation and its usage in the session key generation. The sender can attempt to change the transition key $\lambda_{S}$, if he or she succeeds in cracking the encryption, the same as sender’s private key $D_{SK_{S}}$. Nevertheless, the ttl and new transition value with the attempted hash for the generation of session key $K_{S,R}=\left(\lambda_{S}⊕\;T_{S_{2}}\right)$ will be rejected further on.
- Eavesdropping (man in the middle attack): The attempt of ${\hat{M}}_{u}$ to perform the MITM attack is performed by intercepting reading and modification of message contents. As autonomous protocol consists of encryption/decryption $E_{PK_{S}}$,$\;D_{SK_{S}}$. by user’s public/private key $PK_{S}$, $SK_{S}$ will be very hard to break. Later, the communication event within users is secured by the session key’s $E_{PK_{S}}^{\prime }$, which is unique for every event. These three different groups of keys make the MITM attack fail in the autonomous protocol.
- Replay: The purpose of replay attack is to repeatedly send similar messages with some modification. The public key $PK_{S}$ can be thought of as valid, but the time-stamp $T_{S_{1}}$ needs to be applicable. As discussed earlier, the decryption of the encrypted messages will be a challenge. A small modification will not be beneficial as the response guessing to challenge 1 and 2 would be incorrect. Therefore, a random guessing of the message parameters and replay consisting of time-stamps $T_{S_{1}}$ and $T_{S_{2}}$ will be unacceptable.
- Byzantine: Replaying the intercepted message multiple times and using a nonoptimal path for communication will not be applicable in the autonomous protocol. The replaying will be invalid due to expiry of time-stamps $T_{S_{1}}$ and $T_{S_{2}}$, whereas the nonoptimal path does not exist in the direct communication protocol. Thus, multiple unsuccessful attempts will lead to blocking of the malicious device.
- Location Disclosure: The autonomous protocol process accepts the device/system location only after the successful completion of challenges 1 and 2. The location disclosure attack performs the multiple device location and message exchange recording. As the autonomous systems are dynamic and do not frequently communicate, then the location information will be useless. Additionally, the message recorded will not be useful later due to the update in time-stamps $T_{S_{1}}$, $T_{S_{2}}$, session keys $K_{S,R},\;\;K_{R,S}$, and challenge–response transitions $\lambda_{S}$ and $\lambda_{R}$.

## 7. Experiments

The purpose of the experiments section is to demonstrate the autonomous protocol performance on different devices and the key exchange. A detailed implementation with system settings, session key generation with hash calculations, pseudo-random number generations, protocol performance, and implementation with different time-quarters are explained in detail. To achieve better security and higher performance, the autonomous protocol uses the SHA-256 hash function instead of traditional hash functions. The message exchanges are protected with the cryptographic operations and time limits.

### 7.1. System Configuration

The details of the system configuration used are given in Table 2. The system configuration consisting of different devices shows similar graph behavior with different performance time. The experiments were performed on the separate edge devices: mobile workstation/laptop and Raspberry Pi 3B for measuring the detail performance [31]. We used different PRNG for different time-quarters with no significant difference in the key generation and for performance, but are better for security. In the authentication process, the receiver is responsible for generating the PRNG and sharing it as a challenge matrix *M*_8×8_. Different PRNGs combined with unique time always provide a distinct hash key. The random variables generated by PRNG [32] have higher graph distribution variance and result in good security applications.

### 7.2. Results Analysis

**Figure 4:**
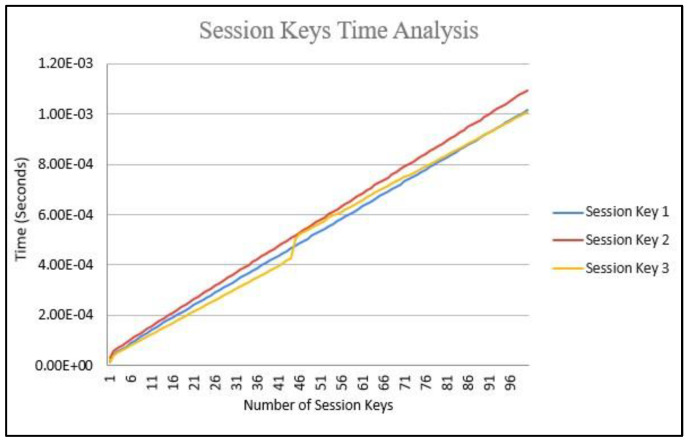
Initial session key time analysis.

In Figure 4, the 100 keys generated for session key 1 $K_{S,R}$, session key 2 $K_{R,S}$, and session key 3 ${{\mathrm{PK}}_{S}}^{\prime }$ are shown. The time required for the calculation on the mobile workstation is seen to be growing at a similar rate amongst the session keys. Figure 4 shows the session-key-generation time analysis, where the time for session key 2 is the highest due to communicating the challenge matrix $M_{8\times 8}$; session key 1 has a similar time to session key 2 for calculating challenge 1, and session key 3 has the lowest time requirements by sharing the final sessional key in the authentication protocol.

**Figure 5:**
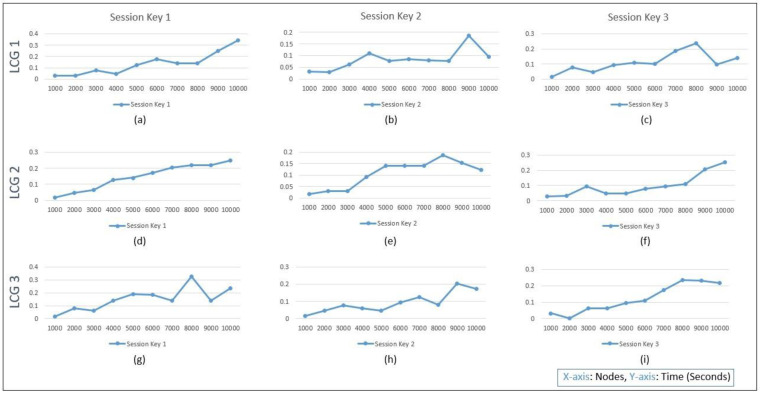
LCG-based session key generation: LCG 1 GCC (**a**) session key 1, (**b**) session key 2, (**c**) session key 3; LCG 2 Borland (**d**) session key 1, (**e**) session key 2, (**f**) session key 3; and LCG 3 Turbo (**g**) session key 1, (**h**) session key 2 and (**i**) session key 3.

The PRNG used within this work refers to the linear congruential generator (LCG) parameters from the standard declared for cryptographic random number preferences [32,33,34]. The session keys generation time in Figure 5 with different LCG parameters can be seen in graph analysis. In the LCG 1 with GCC parameters, the key generation usually grows at an increasing rate because of the computation time requirements, and in (a), (b), and (c), it can be seen that a spike occurs around the 8000 node’s key generation. Similarly, in the case of LCG 2 Borland- and LCG 3 Turbo-based parameters, the graph behavioral analysis spiked after crossing some node limits.

**Figure 6:**
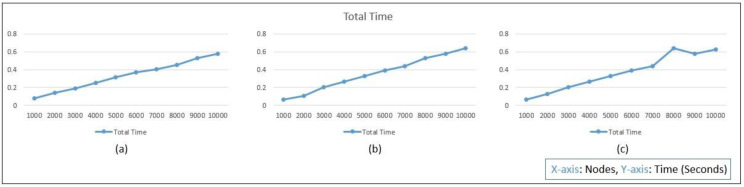
Total time analysis for the LCG: (**a**) GCC, (**b**) Borland, and (**c**) Turbo.

This evaluation can be performed using less computation time if the workstation used is of higher configuration. In Figure 6, the total time required by different LCG parameters is shown by the graph analysis, which is quite similar to each other for 1000 to 10,000 node protocol implementation on the mobile workstation. Even though the parameters are distributed randomly and without any similarity between them, which is known as unpredictable, the time taken by the autonomous protocol implementation in 10,000 nodes is still quite similar.

**Figure 7:**
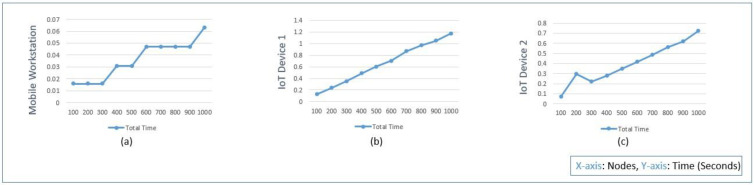
Protocol total time analysis and comparison within the (**a**) mobile workstation, (**b**) IoT device 1, and (**c**) IoT device 2.

In Figure 7, the protocol time analysis for mobile workstation, IoT device 1, and IoT device 2 is shown. The time required on the mobile workstation is less when compared to IoT devices due to the high processing capacity, as shown in Table 2. While increasing the nodes from 100 to 1000, the total time can be seen increasing due to computation requirements for the protocol implementation. In comparison, the protocol implementation on the Raspberry Pi IoT device can be seen increasing smoothly due to less computation capacity. Therefore, the total time is smoother in the low-end device but has higher time implementation requirements on the resource-constrained devices.

**Figure 8:**
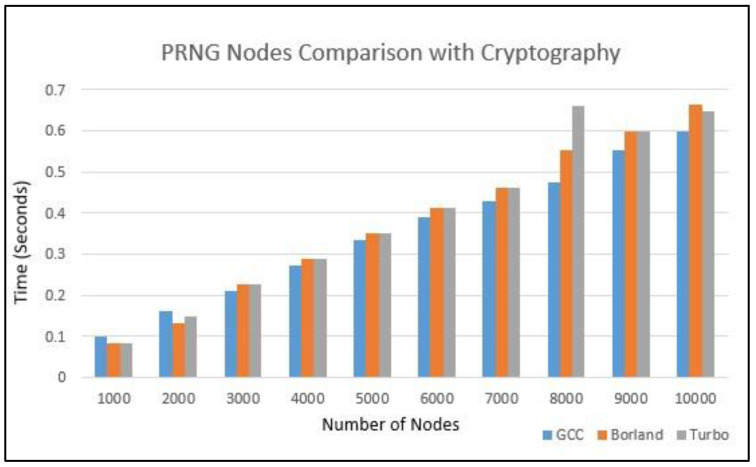
Total time for PRNG-based nodes comparison with cryptography.

Figure 8 shows the comparison of different PRNG parameter settings for the autonomous-protocol-based authentication for the nodes from 1000 to 10,000. As the pseudo-random numbers used here are highly variable in their numerical range, it can be observed in the graph that the time requirements for every one-thousand-node authentication changes in every computation event. Here, the channel encryption is used in such a way that the nodes are generated in parallel so that cryptographic time operation is considered to be in parallel time for every event. The cryptography for every encryption/decryption for complete protocol is performed in 22 milliseconds, referred from the cipher suite 1, consisting of the LEA, SPONGENT, and HMAC algorithms [35]. Several cryptographic operations can be applied, such as lightweight, low latency, and optimal power communication network, for the HLCAS sensor [36], enhanced energy-efficient cryptography method E^3^LCM [37], and resource-constrained device networks, i.e., LED, TWINE, and LEA for 32-bit microcontrollers [38].

Table 3 provides the authentication time between the mobile workstation and IoT device with different LCG parameter settings [32]. GCC LCG parameters are observed to require the highest time for authentication, followed by Borland and Turbo. The time requirements may change on each protocol execution depending on the parameters and time. In the case of IoT device 1 to IoT device 2 authentication, given in Table 4, it is observed that computation time of session key 1 is quite high when compared to the session key 2 and session key 3 time. Similarly, in this case, the total time for GCC is higher when compared to Borland and Turbo. It can be noted that IoT devices require significant time to initialize the protocol when compared to other keys calculation. Table 5 presents the computational cost with parameters for hash function $CC_{h}$ and cryptographic operations $CC_{c}$ for encryption and decryption usage. The total cost provides an analysis for the functions used within the respective protocols.

In Table 6, the feature comparison between multiple protocols is stated. It presents the achievements of the autonomous protocol over recent works. 

In Table 7, the comparison with multiple schemes includes parameters $T_{H}$ as one-way hash function, $T_{ECM}$ as ECC scalar multiplication, $T_{fe}$ as fuzzy extraction operator, and $T_{SE/D}$ as symmetric encryption and decryption [49,50,51].

In Table 8, the total time for protocol completion including communication time is presented. The two IoT schemes referenced, which have efficient mutual authentication [46] and two-phase authentication protocol in WSN [49], suffer due to the inclusion of a server/third party for the certificate-based authentication. In comparison, the autonomous protocol performs much better by excluding the dependency on server/third-party systems for the authentication. Furthermore, in comparison to the referenced systems, the calculation time for the autonomous protocol is also less.

## 8. Conclusions

Autonomous authentication is crucial for secure communication in edge networks. Thus, competitive world-class research is presented that can prevent major attacks, independent of the server/third-party systems and no requirements for storing secret keys, and has better computational time on mobile workstations and resource-constrained IoT devices. The significant idea of the challenge–response system that uses a unique session key for every new authentication is highlighted in this work. The applications present in handheld devices, automobiles, drones, IoT devices work efficiently by applying this protocol. The authentication time on the mobile workstation for parallel 10,000 nodes is around 0.7 s. Furthermore, the time between mobile workstation authentication to IoT mode with the Turbo compiler is 0.094 s, as presented, which is similar to the authentication time between IoT device 1 and IoT device 2. Various experiments prove the effectiveness of the autonomous protocol and its usage in a real-world scenario. In the future, GPU-based IoT device utilization for the parallelization of the exclusive challenge–response scheme will be utilized.

## Figures and Tables

**Table 1:** Notation.

Notation	Meaning
*S*	Sender
*R*	Receiver
*AS*	Registration Server
$a_{x}$	Device Address/Unique Identity (UID)
ttl	Validity of the message/time to live
$t_{x}$	Timestamp of *x*
$PK_{x}$	Public key of *x*
$SK_{x}$	Private key of *x*
$\lambda_{x}$	Transition taken by *x*
$E_{x}$	Encryption performed by *x*’s public key
$D_{x}$	Decryption performed by *x*’s private key
$K_{x,y}$	Session key from *x* to *y*
${\hat{M}}_{x}$	Malicious user *x*
⊕	XOR bitwise operator

**Table 2:** System configuration.

**System**	Mobile Workstation (Laptop)	IoT Device 1	IoT Device 2
**Model**	MacBook Pro	Raspberry Pi 3B	Raspberry Pi 3B+
**Processor**	Intel Core i7 @ 2.6 GHz	Broadcom Quad Core @ 1.2GHz	Broadcom, Cortex-A53 (ARMv8) 64-bit SoC @ 1.4GHz
**Main Memory**	16 GB	1 GB SDRAM	1GB LPDDR2 SDRAM
**Programming Language**	Python 2.7 (Libraries: random, hashlib, datetime, sys, pandas, base64, and getnode.)		

**Table 3:** LCG-based mobile workstation to IoT device 1 authentication time.

LCG	GCC	Borland	Turbo
**Total Time (s)**	0.14100	0.11000	0.09400

**Table 4:** LCG-based IoT device 1 to IoT device 2 authentication time (s).

LCG/Key	Session Key 1	Session Key 2	Session Key 3	Total Time
GCC	0.548990965	0.000441074	0.001973867	0.551431894
Borland	0.089758873	0.001000166	0.00190115	0.092696905
Turbo	0.41444993	0.000444174	0.001885176	0.41680479

**Table 5:** Protocol computation cost comparison.

Reference	User Device	Gateway	Sensor Node	Total
Cloud–IoT [39]	$11*CC_{h}$	$7*CC_{h}$	$5*CC_{h}$	$23*CC_{h}$
Ad hoc wireless sensors [40]	$7*CC_{h}$	$5*CC_{h}$	$7*CC_{h}$	$19*CC_{h}$
Edge–IoT [41]	$9*CC_{h}$	$15*CC_{h}$	$8*CC_{h}$	$32*CC_{h}$
IoT devices [42]	$4*CC_{h}$	$3*CC_{h}$	$2*CC_{h}$	$9*CC_{h}$
Autonomous protocol	$2*CC_{h}+3*CC_{C}$	N.A.	$2*CC_{h}+3*CC_{C}$	$4*CC_{h}+6*CC_{C}$

**Table 6:** Performance metric comparison of IoT security protocols.

Performance Metric	[43]	[44]	[45]	[46]	[47]	[48]	Autonomous Protocol
Number of messages exchanged	8	9	3	6	11	7	4
Security of message exchanged	Y	-	-	Y	N	Y	Y
IoT Device computation efficiency	N	N	N	Y	N	N	Y
Lightweight cryptography	N	Y	N	Y	N	N	Y
Autonomous authentication	N	N	N	N	N	N	Y
Automated key exchange	Y	_	Y	Y	-	N	Y
High-range authentication statistics	N	N	N	N	N	Y	Y
Infrastructure independent	N	N	N	N	N	N	Y

**Table 7:** Computational overhead in IoT schemes.

Reference	User Device	IoT Device	Gateway	Total Time (ms)
[49]	$8T_{H}+4T_{ECM}=70.96$	$10T_{H}+11T_{ECM}=191.3$	$5T_{H}+8T_{ECM}=141.6$	403.86
[45]	$1T_{fe}+5T_{H}+5T_{ECM}=104.2$	$3T_{H}+4T_{ECM}=70$	$4T_{H}+5T_{ECM}=86.78$	260.98
[50]	$9T_{H}+2T_{ECM}=37.08$	$5T_{H}+2T_{ECM}=35.8$	$7T_{H}=2.24$	75.12
[51]	$2T_{H}+1T_{ECM}+2T_{SE/D}=28.94$.	$1T_{H}+1T_{SM}+2T_{SE/D}=28.62$	$2T_{H}+4T_{SE/D}=23.04$	80.6
Autonomous protocol	$2T_{H}+3T_{SE/D}=$ 4.15	$2T_{H}+3T_{SE/D}=$ 11.22	N.A.	15.37

**Table 8:** Total time for the protocol completion.

Reference	Total Time (s)
Efficient mutual authentication scheme in IoT [46]	2.01304
Two-phase authentication protocol in WSN [49]	27.27
Autonomous protocol—mobile workstation to IoT device	0.09400
Autonomous protocol—IoT device 1 to IoT device 2	0.092696905

## Data Availability

Not applicable.

## References

[1] Strand M., Wiik J.H. (2022). Security for Autonomous and Unmanned Devices: Cryptography and its Limits. Anti-Tamper Protective Systems for NATO Operations.

[2] Sucasas V., Mantas G., Saghezchi F.B., Radwan A., Rodriguez J. (2016). An autonomous privacy-preserving authentication scheme for intelligent transportation systems. Comput. Secur..

[3] Chow M.C., Ma M., Pan Z. (2021). Attack models and countermeasures for autonomous Vehicles. Intelligent Technologies for Internet of Vehicles.

[4] Pham M., Xiong K. (2021). A survey on security attacks and defense techniques for connected and autonomous vehicles. Comput. Secur..

[5] Martínez-Cruz A., Ramírez-Gutiérrez K.A., Feregrino-Uribe C., Morales-Reyes A. (2021). Security on in-vehicle communication protocols: Issues, challenges, and future research directions. Comput. Commun..

[6] Aloqaily M., Hussain R., Khalaf D., Hani D., Oracevic A. (2022). On the role of futuristic technologies in securing UAV-supported autonomous vehicles. IEEE Consumer Electronics Magazine.

[7] Nikitas A., Parkinson S., Vallati M. (2022). The deceitful connected and autonomous vehicle: Defining the concept, contextualising its dimensions and proposing mitigation policies. Transp. Policy.

[8] Çakal K., İlker K.A., Aydos M. (2021). Cyber Security of Connected Autonomous Vehicles. Avrupa Bilim Ve Teknol. Derg..

[9] Sun X., Yu F.R., Zhang P. (2022). A Survey on Cyber-Security of Connected and Autonomous Vehicles (CAVs). IEEE Trans. Intell. Transp. Syst..

[10] Nayak B.P., Hota L., Kumar A., Turuk A.K., Chong P.H.J. (2022). Autonomous Vehicles: Resource Allocation, Security, and Data Privacy. IEEE Trans. Green Commun. Netw..

[11] Paruchuri V., Durresi A., Kannan R., Iyengar S.S. Authenticated autonomous system traceback. Proceedings of the 18th International Conference on Advanced Information Networking and Applications, 2004. AINA 2004.

[12] Huang C., Lu R., Lin X., Shen X. (2018). Secure automated valet parking: A privacy-preserving reservation scheme for autonomous vehicles. IEEE Trans. Veh. Technol..

[13] Liang W., Ning Z., Xie S., Hu Y., Lu S., Zhang D. (2021). Secure fusion approach for the internet of things in smart autonomous multi-robot systems. Inf. Sci..

[14] Lapeyre S., Valette N., Merandat M., Flottes M.L., Rouzeyre B., Virazel A. A lightweight, plug-and-play and autonomous JTAG authentication IP for secure device testing. Proceedings of the 2022 IEEE European Test Symposium (ETS).

[15] Wickström J., Westerlund M., Pulkkis G. Smart contract based distributed IoT security: A protocol for autonomous device management. Proceedings of the 2021 IEEE/ACM 21st International Symposium on Cluster, Cloud and Internet Computing (CCGrid).

[16] Alkhalaf S. (2021). A control-driven autonomous authentication scheme for peer-to-peer control systems assisted industrial Internet of things. Soft Comput..

[17] Wang M., Rui L., Yang Y., Gao Z., Chen X. (2022). A blockchain-based multi-CA cross-domain authentication scheme in decentralized autonomous network. IEEE Transactions on Network and Service Management.

[18] Khan M.Z., Sarkar A., Ghandorh H., Driss M., Boulila W. (2022). Information fusion in autonomous vehicle using artificial neural group key synchronization. Sensors.

[19] Li T., Onodera Y., Nakayama Y., Hisano D. Multi-Channel Authentication for Secure D2D using Optical Camera Communication. Proceedings of the 2022 IEEE 19th Annual Consumer Communications & Networking Conference (CCNC).

[20] Yang J., Gope P., Cheng Y., Sun L. (2021). Design, analysis and implementation of a smart next generation secure shipping infrastructure using autonomous robot. Comput. Netw..

[21] Bilbao-Arechabala S., Jorge-Hernandez F. (2021). Security Architecture for Swarms of Autonomous Vehicles in Smart Farming. Appl. Sci..

[22] Jha S., Jha N., Prashar D., Ahmad S., Alouffi B., Alharbi A. (2022). Integrated IoT-based secure and efficient key management framework using hashgraphs for autonomous vehicles to ensure road safety. Sensors.

[23] Bagheri H., Noor-A-Rahim M., Liu Z., Lee H., Pesch D., Moessner K., Xiao P. (2021). 5G NR-V2X: Toward connected and cooperative autonomous driving. IEEE Commun. Stand. Mag..

[24] Rahmani L., Minarsch D., Ward J. Peer-to-peer autonomous agent communication network. Proceedings of the 20th International Conference on Autonomous Agents and MultiAgent Systems.

[25] Lounis K., Zulkernine M. (2021). T2T-MAP: A PUF-based thing-to-thing mutual authentication protocol for IoT. IEEE Access.

[26] Ko T., Ji C., Hong M. (2021). AVoD: Advanced Verify-on-Demand for efficient authentication against DoS attacks in V2X communication. Secur. Commun. Netw..

[27] Duan X., Yan H., Tian D., Zhou J., Su J., Hao W. (2021). In-Vehicle CAN Bus Tampering Attacks Detection for Connected and Autonomous Vehicles Using an Improved Isolation Forest Method. IEEE Transactions on Intelligent Transportation Systems.

[28] Shahzad A., Gherbi A., Zhang K. (2022). Enabling Fog–Blockchain Computing for Autonomous-Vehicle-Parking System: A Solution to Reinforce IoT–Cloud Platform for Future Smart Parking. Sensors.

[29] Wu B., Wu Q., Ying Z. (2022). GAP-MM: 5G-Enabled Real-Time Autonomous Vehicle Platoon Membership Management Based on Blockchain. Secur. Commun. Netw..

[30] Kumar M.S., Vimal S., Jhanjhi N.Z., Dhanabalan S.S., Alhumyani H.A. (2021). Blockchain based peer to peer communication in autonomous drone operation. Energy Rep..

[31] Pismenny B., Eran H., Yehezkel A., Liss L., Morrison A., Tsafrir D. Autonomous NIC offloads. Proceedings of the 26th ACM International Conference on Architectural Support for Programming Languages and Operating Systems.

[32] Pardeshi M.S., Yuan S.M. (2019). SMAP fog/edge: A secure mutual authentication protocol for fog/edge. IEEE Access.

[33] Schneier B. (1996). Applied Cryptography.

[34] Easttom C. (2016). Modern Cryptography: Applied Mathematics for Encryption and Information Security.

[35] Morales-Sandoval M., De-La-Parra-Aguirre R., Galeana-Zapién H., Galaviz-Mosqueda A. (2021). A three-tier approach for Lightweight data security of body area networks in E-health applications. IEEE Access.

[36] Prakasam P., Madheswaran M., Sujith K.P., Sayeed M.S. (2022). Low Latency, Area and Optimal Power Hybrid Lightweight Cryptography Authentication Scheme for Internet of Things Applications. Wirel. Pers. Commun..

[37] Prakasam P., Madheswaran M., Sujith K.P., Sayeed M.S. (2021). An enhanced energy efficient lightweight cryptography method for various IoT devices. ICT Express.

[38] Thakor V.A., Razzaque M.A., Khandaker M.R. (2021). Lightweight cryptography algorithms for resource-constrained IoT devices: A review, comparison and research opportunities. IEEE Access.

[39] Sharma G., Kalra S. (2019). A lightweight user authentication scheme for cloud-IoT based healthcare services. Iran. J. Sci. Technol. Trans. Elect. Eng..

[40] Turkanovic M., Brumen B., Hölbl M. (2014). A novel user authentication and key agreement scheme for heterogeneous ad hoc wireless sensor networks, based on the Internet of Things notion. Ad Hoc Netw..

[41] Wazid M., Das A.K., Shetty S., Rodrigues J.J.P.C., Park Y. (2019). LDAKM-EIoT: Lightweight device authentication and key management mechanism for edge-based IoT deployment. Sensors.

[42] Masud M., Gaba G.S., Choudhary K., Hossain M.S., Alhamid M.F., Muhammad G. (2021). Lightweight and anonymity-preserving user authentication scheme for IoT-based healthcare. IEEE Internet Things J..

[43] Goworko M., Wytrębowicz J. (2021). A secure communication system for constrained IoT devices—Experiences and recommendations. Sensors.

[44] Bala D.Q., Maity S., Jena S.K. Mutual authentication for IoT smart environment using certificate-less public key cryptography. Proceedings of the 2017 Third International Conference on Sensing, Signal Processing and Security (ICSSS).

[45] Challa S., Wazid M., Das A.K., Kumar N., Reddy A.G., Yoon E.J., Yoo K.Y. (2017). Secure signature-based authenticated key establishment scheme for future IoT applications. IEEE Access.

[46] Alshawish I., Al-Haj A. (2022). An efficient mutual authentication scheme for IoT systems. J. Supercomput..

[47] Liu X., Zhao M., Li S., Zhang F., Trappe W. (2017). A security framework for the Internet of Things in the future internet architecture. Future Internet.

[48] Park N., Kang N. (2016). Mutual authentication scheme in secure Internet of Things technology for comfortable lifestyle. Sensors.

[49] Porambage Q.P., Schmitt C., Kumar P., Gurtov A., Ylianttila M. Two-phase authentication protocol for wireless sensor networks in distributed IoT applications. Proceedings of the IEEE Wireless Communications and Networking Conference (WCNC).

[50] Chang C.-C., Le H.-D. (2016). A provably secure, efficient, and flexible authentication scheme for ad hoc wireless sensor networks. IEEE Trans. Wirel. Commun..

[51] Sadhukhan D., Ray S., Biswas G.P., Khan M.K., Dasgupta M. (2021). A lightweight remote user authentication scheme for IoT communication using elliptic curve cryptography. J. Supercomput..

